# A randomized controlled safety and feasibility trial of floatation-REST in anxious and depressed individuals

**DOI:** 10.1371/journal.pone.0286899

**Published:** 2024-06-06

**Authors:** McKenna M. Garland, Raminta Wilson, Wesley K. Thompson, Murray B. Stein, Martin P. Paulus, Justin S. Feinstein, Sahib S. Khalsa

**Affiliations:** 1 Laureate Institute for Brain Research, University of Tulsa, Tulsa, Oklahoma, United States of America; 2 Kendall College of Arts and Sciences, University of Tulsa, Tulsa, Oklahoma, United States of America; 3 Department of Psychiatry, University of California San Diego, La Jolla, California, United States of America; 4 Herbert Wertheim School of Public Health and Human Longevity Science, University of California San Diego, La Jolla, California, United States of America; 5 Psychiatry Service, Veterans Affairs San Diego Healthcare System, San Diego, California, United States of America; 6 Oxley College of Health Sciences, University of Tulsa, Tulsa, Oklahoma, United States of America; 7 Float Research Collective, Kihei, Hawaii, United States of America; University College London, UNITED KINGDOM

## Abstract

**Background:**

Reduced Environmental Stimulation Therapy via floatation (floatation-REST) is a behavioral intervention designed to attenuate exteroceptive sensory input to the nervous system. Prior studies in anxious and depressed individuals demonstrated that single sessions of floatation-REST are safe, well-tolerated, and associated with an acute anxiolytic and antidepressant effect that persists for over 48 hours. However, the feasibility of using floatation-REST as a repeated intervention in anxious and depressed populations has not been well-investigated.

**Methods:**

In this single-blind safety and feasibility trial, 75 individuals with anxiety and depression were randomized to complete six sessions of floatation-REST in different formats: pool-REST (weekly 1-hour float sessions), pool-REST preferred (float sessions with flexibility of duration and frequency), or an active comparator (chair-REST; weekly 1-hour sessions in a Zero Gravity chair). Feasibility (primary outcome) was assessed via an 80% rate of adherence to the assigned intervention; tolerability via study dropout and duration/frequency of REST utilization; and safety via incidence of adverse events and ratings about the effects of REST.

**Results:**

Of 1,715 individuals initially screened, 75 participants were ultimately randomized. Six-session adherence was 85% for pool-REST (mean, *M* = 5.1 sessions; standard deviation, *SD* = 1.8), 89% for pool-REST preferred (*M* = 5.3 sessions; *SD* = 1.6), and 74% for chair-REST (*M* = 4.4 sessions; *SD* = 2.5). Dropout rates at the end of the intervention did not differ significantly between the treatment conditions. Mean session durations were 53.0 minutes (*SD* = 12.3) for pool-REST, 75.4 minutes (*SD* = 29.4) for pool-REST preferred, and 58.4 minutes (*SD* = 4.3) for chair-REST. There were no serious adverse events associated with any intervention. Positive experiences were endorsed more commonly than negative ones and were also rated at higher levels of intensity.

**Conclusions:**

Six sessions of floatation-REST appear feasible, well-tolerated, and safe in anxious and depressed individuals. Floatation-REST induces positively-valenced experiences with few negative effects. Larger randomized controlled trials evaluating markers of clinical efficacy are warranted.

**Clinical Trial Registration Identifier:**

NCT03899090.

## Introduction

Depression and anxiety-related disorders are widespread, with lifetime prevalence rates estimated at 21% and 29%, respectively [1], and they exert substantial distress and dysfunction at individual and societal levels [2]. Depression and anxiety can occur in isolation, but clinical experience indicates that comorbidity of these disorders is more often the rule rather than the exception, with an estimated comorbidity rate ranging between 50 and 90% [3–5]. The consequence of such comorbidity is a synergistic dysfunction marked by greater disability (i.e., higher levels of interpersonal and occupational dysfunction) and poorer outcomes (i.e., higher mortality rates) compared to anxiety or depression alone [4]. Despite the range of currently available treatment modalities, including pharmacotherapy, psychotherapy, or their combination, there is great variability in whether individuals with depression or anxiety will reach remission status. Response rates range from 8–50% depending on the treatment modality [6, 7], with lower response rates and worse treatment outcomes in individuals with comorbid depression and anxiety [3, 8, 9]. Given these substantial challenges, the exploration of additional treatment interventions is needed.

Reduced Environmental Stimulation Therapy via floatation (i.e., floatation-REST) is a behavioral intervention designed to systematically attenuate exteroceptive sensory input to the nervous system. The most common form, floatation-REST, involves floating effortlessly in a shallow pool of water heated to skin temperature and saturated with Epsom salt to increase buoyancy (consequently, it is sometimes also called ‘pool-REST’). The environment is specially engineered to be lightproof, soundproof, and humidity and temperature-controlled, so that input from visual, auditory, olfactory, gustatory, thermal, tactile, vestibular, and proprioceptive channels is minimized, as is movement and speech [10]. Chair-REST has also emerged as an alternative form of REST [11, 12]. In this environment, individuals recline in an ergonomically engineered chair designed to take pressure off the spinal cord. Typically, these chairs are placed in dimly lit and quiet rooms, producing similar but not identical environments to those experienced during pool-REST.

Insight into the utility of REST as a clinical intervention for anxious and depressed individuals is currently limited. One of the most common observations in non-clinical and clinical samples is that REST appears to induce acute (i.e., short-term) anxiolytic and antidepressant effects [10, 12–19]. REST has also been associated with acute reductions in muscle tension and pain [10, 14–16, 20–23]; this finding is relevant as muscle tension and pain represent somatic symptoms commonly reported by anxious and depressed individuals [24]. However, there has been limited documentation regarding the safety, feasibility, and tolerability of REST. In our initial studies examining the effects of single sessions of pool-REST or chair-REST in clinically anxious and depressed individuals, we observed no adverse events, suggesting that single session exposure was safe and well-tolerated [10, 12]. However, no studies have systematically evaluated the safety profile of this intervention across multiple sessions in anxious and depressed populations. It is important to note that there have been several deaths reported with the use of floatation tanks at recreational float centers associated with concurrent drug (i.e., ketamine) or alcohol use [25], suggesting the need for inclusion of substance use screening. There have also been reports of auditory and visual hallucinations during floatation-REST [26]. However, these are generally described in a positive light, and are infrequent [10, 27]. Other negative events that have been reported include occasional skin itchiness or dry mouth, as well as discomfort resulting from accidental introduction of saltwater to the eyes or open wounds [10, 28]. Although no studies have directly evaluated the feasibility of REST as a primary outcome, a wait-list control trial reported that individuals scoring high on self-reported measures of generalized anxiety were largely adherent to 12 sessions of floatation-REST [17]. Another study found that individuals with stress-related pain and burnout depression were able to complete multiple sessions of floatation-REST (in some cases as many as 33 sessions) [16]. However, neither of these studies reported on adverse events that could have arisen during the repeated sessions of floatation-REST. In addition, in prior studies, most anxious individuals were able to tolerate fixed durations of pool-REST or chair-REST (ranging between 45 to 90 minutes) [10, 12, 13, 17], but the impact of flexible assignment on preference (for both session duration and inter-session intervals) has not been investigated.

The current study examined the 1) feasibility, 2) tolerability, and 3) safety of floatation-REST as a repeatable intervention in clinically anxious and depressed individuals using a randomized design with three conditions: pool-REST (1-hour sessions in a floatation pool), pool-REST preferred (sessions in a floatation pool but participants allowed to select their preferred frequency and duration of the float sessions), and an active comparator (chair-REST; 1-hour sessions in a Zero Gravity chair). For our primary outcome of feasibility, we hypothesized that feasibility would be reflected by 6-session adherence rates of 80% or above, a level similar to standard behavioral interventions for anxiety and depression (Hypothesis 1a) [29, 30]. As an additional feasibility assessment, we evaluated the credibility and expectancy of the assigned REST interventions for potential differences (Hypothesis 1b). Regarding tolerability, we predicted that all forms of floatation-REST would be well-tolerated, with low dropout rates (<20%) over the course of the entire intervention and 6-month follow-up period (Hypothesis 2a). We also predicted that individuals would remain in their assigned REST environment for the majority of the allotted session duration (Hypothesis 2b). We also predicted that the duration of float sessions in the pool-REST preferred group would be longer than the other groups based on results from a prior study showing that half the sample wanted to float for longer than 60 minutes [10] (Hypothesis 2c). Finally, based on the prior literature, we predicted that repeated sessions of REST would be safe and associated with minimal adverse reactions (Hypothesis 3).

## Methods

### Participant recruitment

75 treatment-seeking adults with anxiety and depression were recruited through the Laureate Institute for Brain Research’s T1000 and CoBRE databases [31] and from the local community (see below for sample size determination). Inclusion criteria included high levels of current anxiety (as measured by an OASIS score ≥ 6; [32]) and anxiety sensitivity (as measured by an ASI-3 score ≥ 24; [33]). Of note, the initial eligibility criteria of OASIS score ≥ 8 and ASI-3 score ≥ 29 were lowered in September 2020 to increase participant enrollment to meet the specified recruitment rate during the COVID-19 pandemic, a change that was implemented with approval from the funding agency. The rationale for selecting an OASIS cutoff of 6 was based on the following: a cutoff score of 5 was found to separate anxious from non-anxious subjects [34] and a cutoff score of 6 was found to indicate an anxiety disorder of moderate severity [35]. Notably, the original OASIS psychometric study [36] stated that a cutoff score of 6 could be used "to identify all possible cases of anxiety disorders”, and after discussion this was considered to be a reasonable compromise to facilitate recruitment of the target population. Depression symptoms were measured via the Patient Health Questionnaire (PHQ-9, [37]), but an inclusion cutoff score was not specified. Other inclusion criteria included: age between 18 and 60 years (based on the age cutoff of 55 in the T1000 and CoBRE databases), and either no prior experience of floatation-REST or a minimum of 1 year since the last float session. If taking psychiatric medications or receiving psychotherapy, the treatment regimen was required to be stable, defined as having taken the medication or been in therapy for 8 weeks or longer. Daily benzodiazepine use was exclusionary. Participants taking benzodiazepines or opioids on an as-needed basis had to be willing to abstain from use within 24 hours of each float session. Other exclusion criteria included feeling uncomfortable being in water or receiving blood draws, any skin conditions or open wounds that could cause pain when exposed to saltwater, diagnosis of a neurological disorder (e.g., epilepsy), a comorbid diagnosis of bipolar disorder, schizophrenia, or an eating disorder, active suicidality with plan or intent, receiving current inpatient psychiatric treatment, or moderate to severe substance use disorder (as determined by the MINI 7.0). Evidence of current drug use was also assessed prior to each floatation-REST session via urine drug screen testing for amphetamines, methamphetamines, cocaine, barbiturates, benzodiazepines, methylenedioxymethamphetamine (MDMA), methadone, opiates, oxycodone, phencyclidine, propoxyphene, and ketamine, and breathalyzer testing for alcohol; a positive readout on any of these tests was exclusionary. Pregnancy was exclusionary based on a positive urine pregnancy test.

Participants were selected from the T1000 and CoBRE cohorts at LIBR based on the inclusion cutoff criteria for the OASIS and ASI-3 detailed above. All participants who met these cutoff criteria were contacted to determine full eligibility and interest. Once this list had been exhausted, we had to recruit additional participants who were naïve to the T1000 and CoBRE studies in order to complete the sample. Approximately half of the sample fell into this category. All study procedures were approved by the ethics committee (Western Institutional Review Board Protocol #20150528; initial approval date February 12, 2018) and were performed in accordance with Declaration of Helsinki. All participants provided their written informed consent prior to participation and were compensated for their study involvement. In addition, the individuals pictured in the figures in this manuscript have provided written informed consent (as outlined in PLOS consent form) to publish these details. The study was pre-registered on ClinicalTrials.gov (NCT03899090).

### Sample size determination

No power analyses were conducted for this early-phase trial, following the requirement of the funding opportunity announcement associated with the grant funding for this study. Sample size was based on our primary outcome (adherence) and was determined by assuming the distribution of individual adherence proportion is approximately symmetric, with a sample size of 25 subjects per group providing an adherence estimate with margin of error: t_0.975,24_/√25 = 0.41 times the standard deviation at 95% confidence. Given the paucity of previous float research in this target population, it was also difficult to accurately estimate the number of individuals who would drop out of the study. We used rates of dropout for psychotherapy studies for anxiety and depression, which have an estimated attrition rate of ~20% (Taylor et al., 2012; Hembree et al., 2003) [38, 39] as a proxy, which would equate to a total of 60 completers (20 participants/condition). Therefore, we estimated that 25 participants per condition should be sufficient for examining basic patterns of adherence.

### Experimental design and procedures

**Fig 1:**
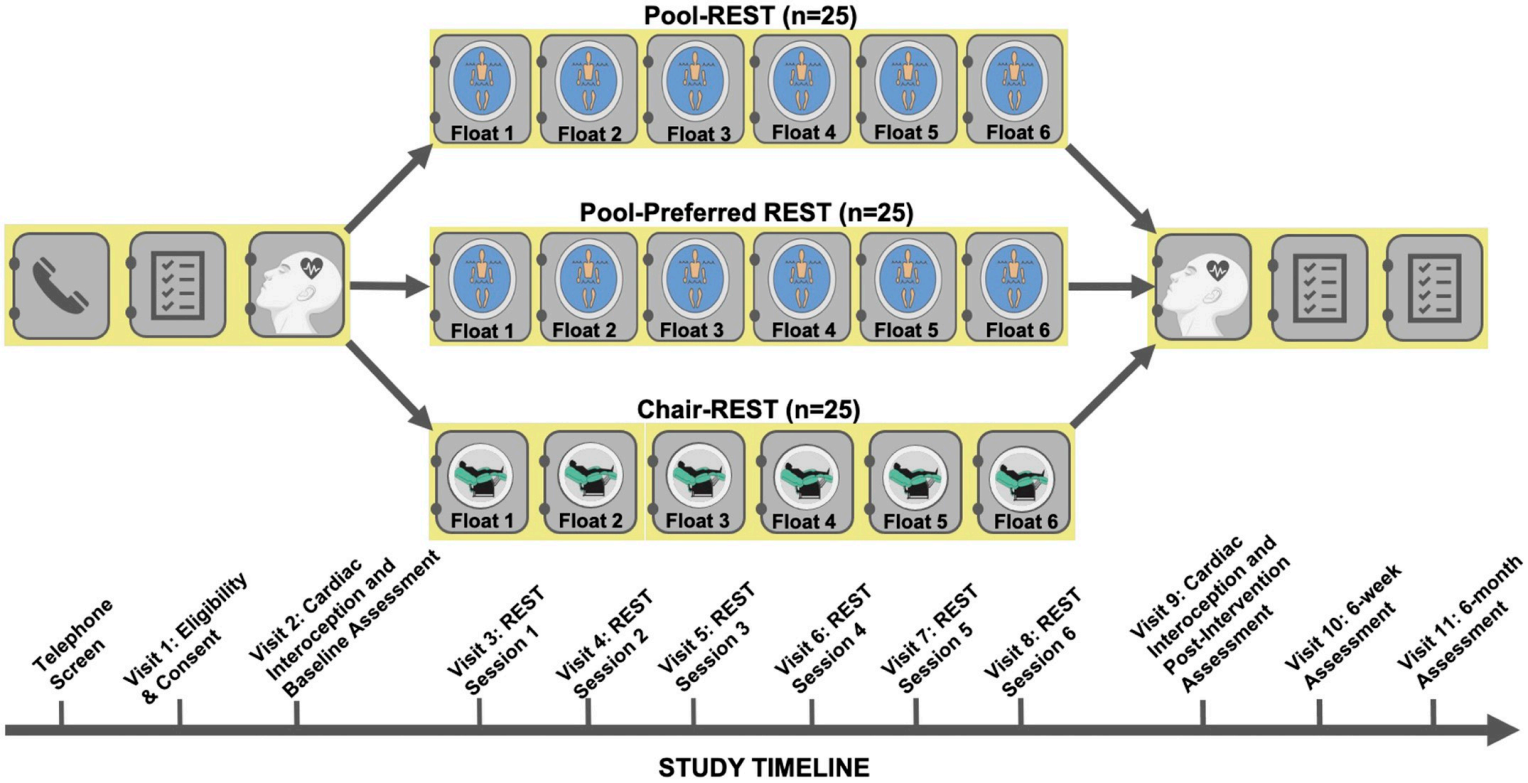
Study visit timeline.

Using a randomized parallel study design, participants completed a total of 11 visits. The study visit timeline is outlined in Fig 1. Participants initially completed a brief telephone screen to collect demographic information and evaluate potential study eligibility. During visit 1, official study eligibility was determined using the inclusion/exclusion criteria. After providing informed consent, participants returned for visit 2, where baseline symptom ratings were completed via clinician-reported and self-reported scales, and behavioral testing of cardiac interoception (results to be reported separately). Visit 2 concluded by unblinding and informing the participant of their randomized assignment (1:1:1) to one of three intervention conditions: pool-REST, pool-REST preferred, or chair-REST (i.e., the active comparator, described further below). The randomization schedule was pre-determined by a statistical consultant uninvolved in the study before participant recruitment and stored as a blinded electronic list. That list was converted to individual envelopes containing allocation information, which were sealed and number-ordered per the sequence of the blinded electronic list. These envelopes were stored in the study coordinator’s office. At the time of assignment, the study coordinator selected the next envelope in the sequence and unblinded and revealed the participant’s allocation (via opening of sealed envelope). At the start of visit 3, all participants completed the Credibility/Expectancy Questionnaire (CEQ; [40]), to assess their pre-intervention attitudes towards the assigned intervention arm. Participants then completed six REST sessions (visits 3–8) according to their assigned condition. We assessed serious and non-serious adverse events after each session via a detailed survey of positive and negatively valenced experiences using a scale drawn from our prior floatation-REST study [10] and via verbal self-report throughout the study period. Following the sixth and final session (visit 8), participants returned to the lab for a post-intervention session (visit 9), where clinician-reported and self-reported symptom ratings were once again collected and behavioral testing of cardiac interoception was completed. The same clinician and self-reported symptom scales were completed again at 6-week (visit 10) and 6-month (visit 11) follow-up visits. Follow-up surveys were initially administered in the laboratory via an electronic tablet, but due to the COVID-19 pandemic these were switched to remote visits starting in May 2020. All surveys were obtained electronically using REDCap (Research Electronic Data Capture; Version 10.6.19; [41, 42]).

### Experimental conditions

#### Pool-REST

**Fig 2:**
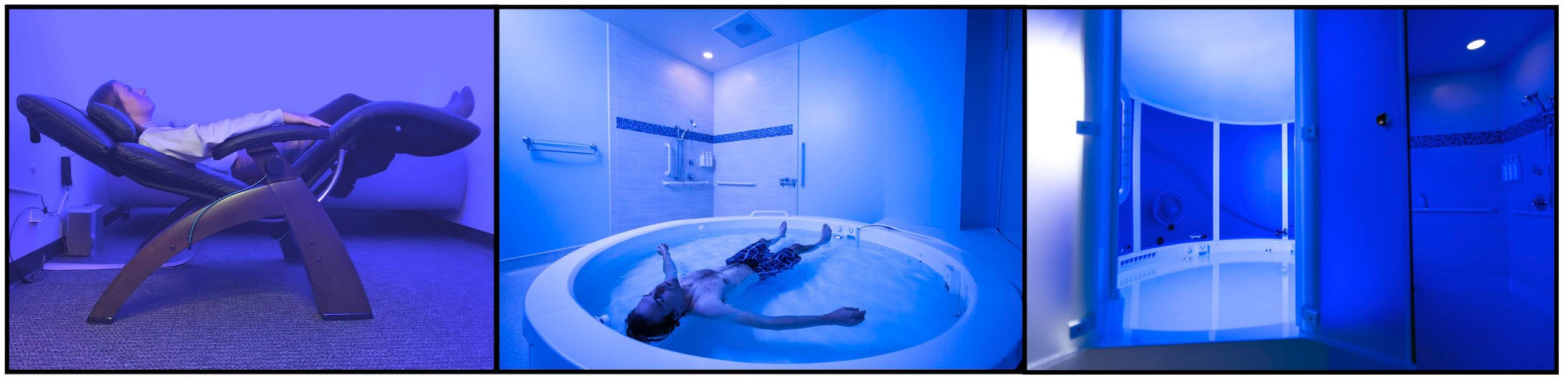
REST Environments utilized in the current study. Pictured from left to right: Chair-REST, Pool-REST in an open pool, and Pool-REST in an enclosed pool. Middle panel of Fig 2 is similar but not identical the original image used in Fig 2 of [10], and is provided to illustrate the different REST environments.

The pool-REST condition, which was delivered at the LIBR facility, involved prescribed (i.e., fixed) session durations and inter-session intervals within an open or enclosed circular float pool (Floataway Inc., Norfolk, United Kingdom, Fig 2). Participants were instructed to complete 1-hour float sessions once per week for six weeks. Each pool measured 2.44 meters in diameter and 0.28 meters in depth. The water in each pool was saturated with over 800 kilograms of Epsom salt (magnesium sulphate), creating a specific gravity of approximately 1.26. This dense saltwater solution allowed for participants to float effortlessly in a supine position. Both pools were constructed in rooms designed to be lightproof and soundproof. The air and water temperature of each room was also calibrated to match the temperature of the skin surface (~35.0 degrees Celsius). More details about how the pool-REST condition was specially engineered to minimize all manner of external sensory stimulation can be found in [10]. We allowed participants to choose between the open and enclosed pool to provide some flexibility for preference and to facilitate the optimal flow of participants through the study.

#### Pool-REST preferred

The pool-REST preferred condition involved increased flexibility of both the session duration and the frequency. Participants were instructed that they were to complete six sessions in the open or closed pool, but they were allowed to freely set their float duration for up to two hours per session. In addition, they were permitted to choose the frequency of their floating schedule, such that they could float as often as they preferred within a 12-week period, with the only requirement being that there needed to be a minimum of 24 hours between sessions.

#### Chair-REST

During chair-REST, which was delivered at the LIBR facility, participants reclined in a Zero Gravity Chair (PC510, Classic Power, Series 2, Human Touch Inc., Long Beach, CA, Fig 2). This active comparator condition was tailored to closely match the pool-REST condition on many parameters including a supine body position, the same prescribed session duration and inter-session interval (i.e., 1-hour sessions completed once per week for six weeks) in a room with reduced light and sound, and a similar instruction set emphasizing the importance of stillness and wakefulness throughout each session (see S1 File for full instruction set). Participants were informed that the chair was ergonomically designed to take pressure off the spinal cord and used memory foam backing to help the chair conform to each participant’s body shape. To match the instruction set across REST conditions, the Zero Gravity chair was explicitly referred to as the “float chair” and the act of lying in the chair was referred to as “floating.” The chair was placed in a dark and quiet room, although the degree of light and sound attenuation was not as strong as that delivered in the pool conditions (i.e., participants could perceive a low-level of external sound and light). Unlike the pool conditions, the ambient temperature was maintained at a normal room temperature of ~23.3 degrees Celsius, so participants randomized to the chair-REST condition remained fully clothed during each session.

### Measures

#### Events assessment

A 43-item self-report measure ([10]; see S1 File) was used to assess safety and subjective experiences occurring during each float session. This assessment was adapted from our previous pilot studies and was designed to assess potential adverse experiences associated with floatation-REST. Twenty nine of the 43 items assessed the presence of negative physical or psychological events including itchiness, dry mouth, pain, anxiety/panic, flashbacks, suicidality, and hallucinations. We also assessed for the presence of positive experiences (e.g., heightened feelings of joy, refreshment, serenity); these 14 items were included to reduce response biases, as well as to prevent a sole focus on assessing negative experiences associated with REST. Each item was rated on four-point scale (“none,” “mild,” “moderate,” or “extreme”) using the following instructions: “*Did you notice or experience an increase in any of these items during or shortly after your float today*? *Please only mark items that showed an increase from your typical day-to-day experience*.” For any item that had an endorsement other than “None,” participants were provided a free-response box to describe their experience in more detail. For certain negative symptoms (strong emotional memories, flashbacks, heightened thoughts related to death, visual or auditory hallucinations, out-of-body experiences, feelings of detachment, loss of control over behavior, and flight of ideas/racing thoughts) any endorsement other than “None” prompted participants with the following question: *“Overall*, *was this a positive or negative experience*?*”* [10]. Items rated as “none” were coded as 0, negatively experienced items ranged from -3 (“extremely negative”) to -1 (“mildly negative”), and positively experienced items ranged from 1 (“mildly positive) to 3 (“extremely positive”).

#### Credibility/Expectancy assessment

The Credibility/Expectancy Questionnaire (CEQ; [40]) was used to measure attitudes about treatment feasibility. The CEQ is a 6-item self-report measure with individual items rated on a 1–9 or a 0%-100% scale. Items assess attitudes about “how believable, convincing, and logical a treatment is” (i.e., credibility; [43]), and expectations one holds about how the treatment might influence symptoms (i.e., expectancy).

There were no changes to the primary outcome or the main secondary outcomes reported in the current study. A number of additional outcomes were specified in May 2022, prior to analysis of the data, and will be included in a separate report.

## Statistical analysis

### Feasibility

The primary outcome of feasibility was assessed by calculating adherence to the assigned sessions via the formula $\hat{p}=\sum_{i=1}^{n}p_{i}/n$, where *p*_*i*_ was the proportion of completed sessions (out of 6) for subject *i* and *n* was the number of subjects per condition [i.e., 25]. To measure attitudinal aspects of feasibility, pre-treatment credibility beliefs were calculated by taking the mean of the first three items of the CEQ [44], while expectancy beliefs were captured via a single item rated on a 0 to 100% scale assessing how much improvement in their symptoms the participant thought would occur after completing the assigned intervention. Two separate one-way ANOVAs were performed to assess for between-group differences in pre-intervention credibility and expectancies, respectively.

### Tolerability

Tolerability was assessed in several ways; first, by calculating the overall dropout rate for each condition (i.e., (number of subjects who withdrew from the study or who were lost to follow-up)/25). Secondly, a Kaplan-Meier analysis with post-hoc log-rank tests was used to test for differential dropout rates over time for the three assigned conditions [45]. Third, within-session tolerability (i.e., session duration in minutes) was modeled with a linear mixed model (estimated using REML and the nloptwrap optimizer) with visit (i.e., visits 1–6 [categorical variable]), condition (i.e., chair-REST, pool-REST, pool-REST preferred), and visit/condition interaction as fixed effects and subject ID as a random effect with an AR1 covariance structure. Post-hoc two-sided t-tests with Holm corrections were used to interpret significant main effects, as well as simple effects for all significant interactions.

Finally, the impact of flexible assignment on preferred frequency of floating for the pool was measured by calculating the mean number of days between float sessions and the corresponding standard deviation. To assess whether session frequency in this condition was related to baseline severity of anxiety, depression, and anxiety sensitivity symptoms, we examined bivariate correlations between float frequency and baseline OASIS, PHQ-9, and ASI-3 scores. Additionally, we examined bivariate correlations between the mean session duration (in minutes) for each intervention and baseline measures of symptom severity (i.e., OASIS, PHQ-9, and ASI-3).

### Safety

The number of adverse and serious adverse events and their relationship to the intervention were recorded over the course of the entire study, including during the follow-up period (see S1 File for definition of serious adverse events). We also tabulated a raw count of the number of negative experiences from the Events Assessment that were rated above mild for each condition (i.e., rated moderately negative or extremely negative). We then fit a linear mixed model (estimated using REML and the nloptwrap optimizer) to predict event magnitude ratings with event, visit, condition, event/visit, condition/visit, and event/visit/condition interactions as fixed effects, and subject ID and visit as random effects, with an AR1 covariance structure. Post-hoc two-sided t-tests with Holm corrections (Holm, 1979) [46] were used to interpret significant main effects, as well as simple effects for all significant interactions. All analyses were performed in RStudio (Version 3.6.0; [47]).

## Results

### Participant characteristics

**Fig 3:**
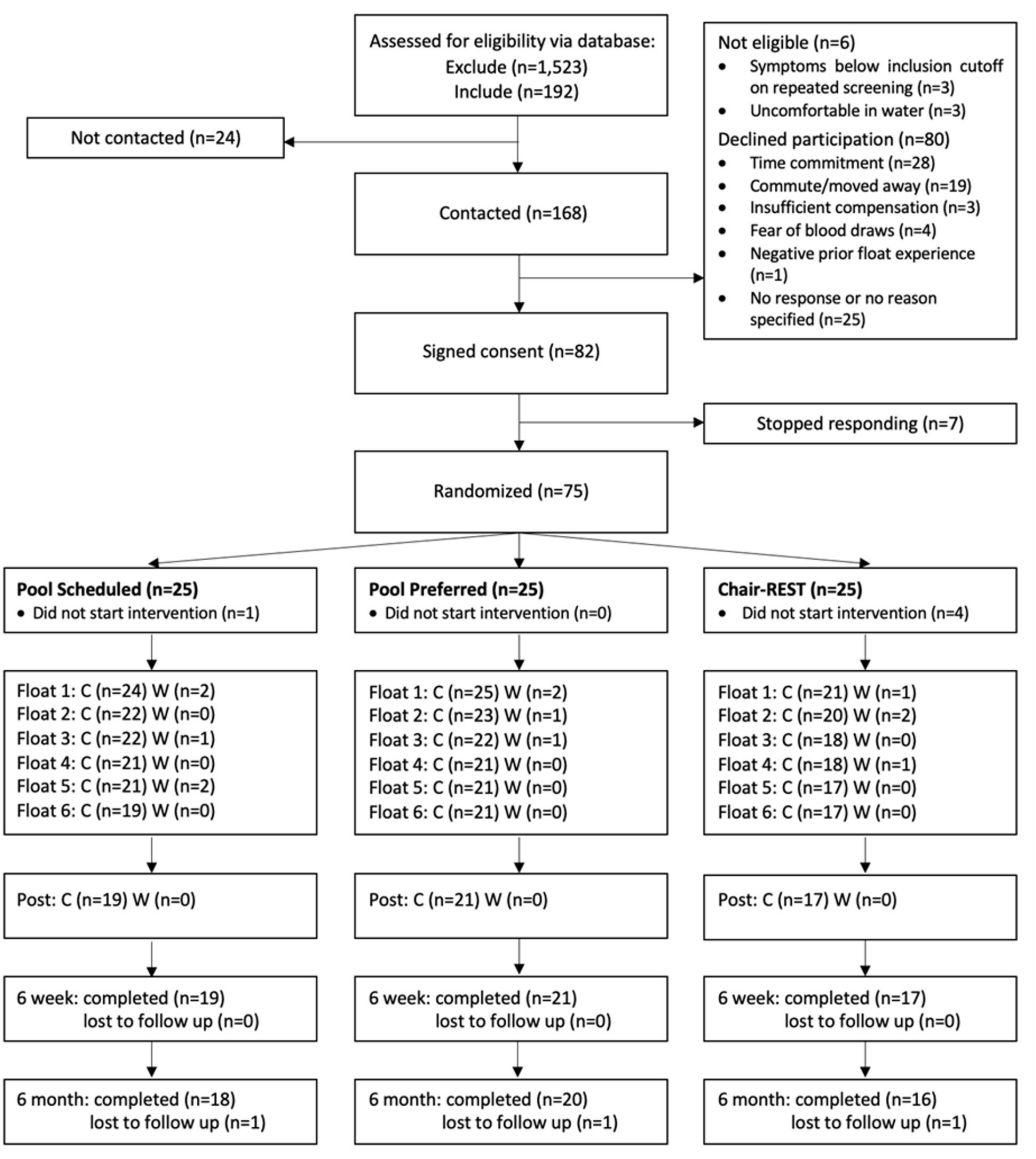
CONSORT diagram. Note. C = Completed. W = Withdrew.

Recruitment occurred between February 2019 and October 2021, with a four month pause starting March 2020 during the COVID-19 pandemic and was terminated upon achievement of the recruitment target. Of the 1,715 individuals screened, 192 met inclusion criteria. We contacted 168 individuals, and 82 individuals consented for participation. Eight individuals stopped responding prior to study initiation, leaving 75 participants who were ultimately randomized. A CONSORT diagram reflecting the flow of participants in each arm is shown in Fig 3. Sociodemographic and clinical characteristics for the 75 individuals who were randomized to each intervention arm are summarized in Table 1. Kruskal-Wallis tests revealed that the groups did not differ significantly on baseline OASIS, ASI-3, or PHQ-9 scores. Most of the sample identified as non-Hispanic White (81%). The most common psychiatric diagnosis in the sample was major depressive disorder (97%) followed by generalized anxiety disorder (51%).

**Table 1:** Participant demographics, DSM-5 diagnostic comorbidities, and screening scores at study entry.

	*Chair-REST* (n = 25)	*Pool-REST* (n = 25)	*Pool-REST preferred* (n = 25)	χ^2^	*df*	*p*
**Demographics**						
Sex						
Male, N (%)	6 (24%)	6 (24%)	5 (20%)			
Female, N (%)	19 (76%)	19 (76%)	20 (80%)			
Age	37.8 (13.4)	33.4 (11.7)	34.3 (9.0)			
Years of Education	15.2 (1.3)	15.1 (1.8)	15.8 (1.7)			
BMI	29.3 (7.3)	28.8 (5.1)	27.9 (6.9)			
Psychiatric Medications^a^	0.7 (1.1)	0.3 (0.6)	1.0 (1.3)			
Receiving Psychotherapy, N(%)	11 (44)	11 (44)	16 (64)			
Race/Ethnicity, N(%)						
White/Caucasian	22 (88)	20 (80)	19 (76)			
Black/African American	2 (1)	6 (24)	4 (16)			
Am. Indian/Alaskan Native	5 (20)	5 (20)	4 (16)			
Asian/Pacific Islander	1 (4)	0 (0)	0 (0)			
Hispanic	1 (4)	2 (8)	2 (8)			
Unspecified	0 (0)	0 (0)	2 (8)			
**Self-Report Measures**						
OASIS Anxiety	10.1 (2.5)	9.4 (2.8)	9.9 (2.5)	1.18	2	0.55
ASI-3 Anxiety Sensitivity	35.6 (13.0)	39.9 (9.4)	40.5 (11.8)	0.14	2	0.93
PHQ-9 Depression	12.5 (4.9)	11.5 (5.5)	12.1 (4.7)	0.95	2	0.62
**Psychiatric Diagnosis**, N(%)^b^						
Major depressive disorder	24 (96)	24 (96)	25 (100)			
Generalized anxiety disorder	10 (40)	17 (68)	11 (44)			
Social anxiety disorder	9 (36)	12 (48)	8 (32)			
Posttraumatic stress disorder	9 (36)	8 (32)	7 (28)			
Panic disorder	3 (12)	6 (24)	7 (28)			

*Note*. Numbers reflect means and standard deviation unless otherwise indicated. BMI = Body Mass Index; OASIS = Overall Anxiety Severity and Impairment Scale [32] measured at pre-intervention visit; ASI-3 = Anxiety Sensitivity Index-3 [33], measured at pre-intervention visit; PHQ-9 = Patient Health Questionairre-9 [37] measured at pre-intervention visit.

^a^Numbers reflect the mean number of prescribed psychiatric medications taken by the participant, measured at the initial screening.

^b^Diagnosis was determined by clinician interview using the MINI version 7.0 [48]. For brevity, only comorbid diagnoses with frequency > 10% across the entire sample (n = 75) are listed. As this study allowed for comorbid diagnoses, percentage totals will be > 100%.

### Primary outcome

#### Feasibility

*Adherence*. Mean treatment adherence was 89% (*M* = 5.3 sessions, *SD* = 1.6) for the pool-REST preferred condition, 85% (*M* = 5.1 sessions, *SD* = 1.8) for the pool-REST condition, and 74% (*M* = 4.4 sessions, *SD* = 2.5 sessions) for the chair-REST condition.

### Secondary outcomes

#### Feasibility

**Fig 4:**
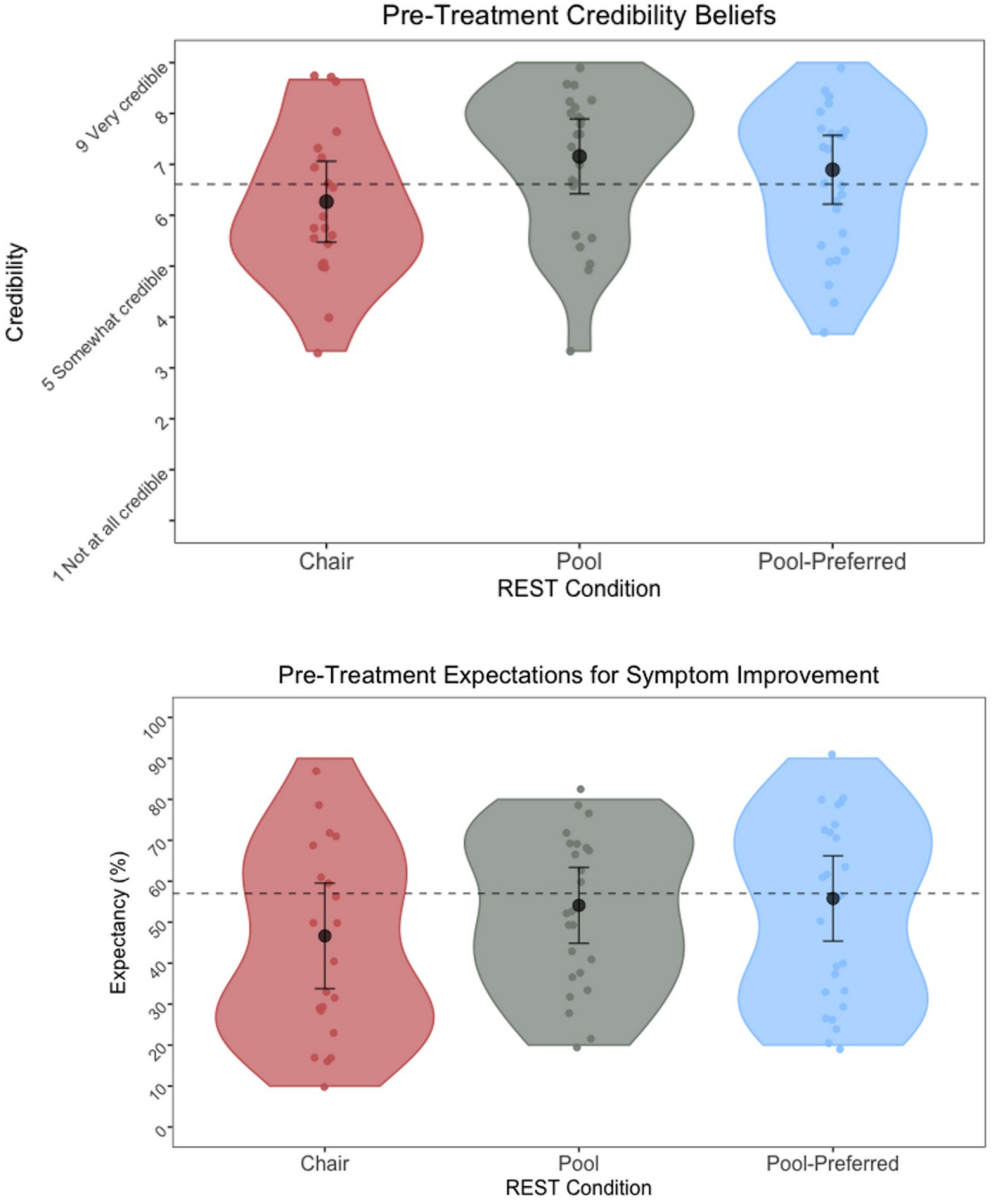
Pre-Treatment credibility beliefs and expectations for symptom improvement. *Note*. Figures reflect pre-intervention ratings for credibility of beliefs (top) and expectations for symptom improvement (bottom) on the Credibility and Expectancy Questionnaire, according to the assigned intervention. The black dots and error bars represent the mean and standard error of the mean, respectively. Grey dashed lines reflect an established mean for credibility and expectancy beliefs of other non-pharmacological treatments (e.g., psychotherapy, see discussion). There were no significant group differences in levels of pre-treatment credibility or expectancy beliefs (*p*s >.05).

*Credibility and expectancy*. One-way ANOVAs revealed that the REST conditions did not differ significantly on pre-intervention CEQ credibility (F(2, 67) = 2.42, *p* = 0.097, Eta^2^ = 0.07, 95%), or expectancy (F(2, 67) = 0.98, *p* = 0.379; see Fig 4). The mean credibility score for the three groups was 6.67 (*SD* = 1.50). Credibility beliefs did not significantly differ between chair-REST and pool-REST (*t* = -2.19, p = 0.10, 95% CI [-2.04, 0.12]), chair-REST and pool-REST preferred (*t* = -1.07, *p* = 0.48, 95% CI [-1.54, 0.60]), or between pool-REST and pool-REST preferred (*t* = 1.19, *p* = 0.48, 95% CI [-0.53, 1.53]). On average, the three groups expected the intervention to halve their symptoms (*M* = 50.3%, *SD* = 21.6%), and expectations did not significantly differ between chair-REST and pool-REST (*t* = -1.32, p = 0.57, 95% CI [-0.25, 0.07]), chair-REST and pool-REST preferred (*t* = -1.25, *p* = 0.57, 95% CI [-0.24, 0.08]), or between pool-REST and pool-REST preferred (*t* = 0.01, *p* = 0.93, 95% CI [-0.15, 0.16]).

#### Tolerability

**Fig 5:**
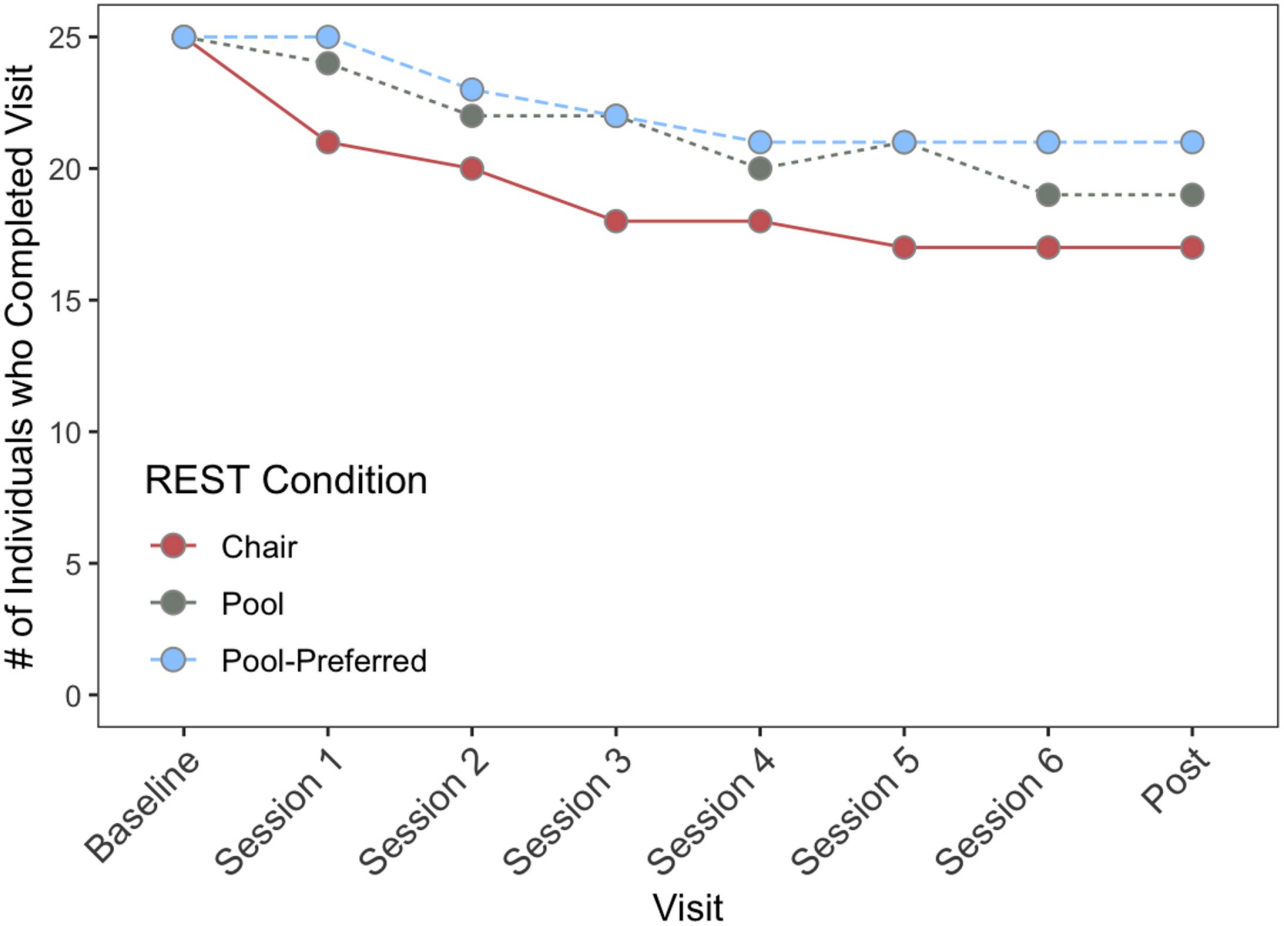
Dropout progression by REST condition. Drop-out percentages at each time point were derived as follows: (number of subjects who withdrew from the study or who were lost to follow-up)/25.

*Dropout*. Following the randomization at the baseline visit, the chair-REST condition had the highest dropout rate (i.e., number of subjects who withdrew from the study or who were lost to follow-up)/25) at 16% (n = 4), versus 4% (n = 1) for the pool-REST condition and 0% for the pool-REST preferred condition (n = 0). Across the six-session intervention, the chair-REST condition demonstrated an overall dropout rate of 32% (n = 8), while the pool-REST condition dropout rate was 24% (n = 6), and the pool-REST preferred dropout rate was 16% (n = 4). The dropout rate at the 6-month follow up was 36% for the chair-REST condition (n = 9), 28% for the pool-REST condition (n = 7), and 20% for the pool-REST preferred condition (n = 5; i.e., one additional individual dropped out from each of the groups). The Kaplan-Meier survival analyses did not indicate a significant difference in treatment dropout among the three groups (*p* = .40, Fig 5).

Of note, due to the onset of the COVID-19 pandemic and mandatory shutdowns, two participants were withdrawn from the study by the study investigators, as the participants were unable to complete their six REST sessions within the prespecified inter-session intervals. One participant in the Chair-REST condition was withdrawn from the study after session 2 (at day 27), and one participant from the Pool-REST condition was withdrawn following their fifth session (at day 73). This dropout information was entered into the analysis, but due to the small amount of dropout and no discernable group pattern, no further corrective action was taken during the analysis.

**Fig 6:**
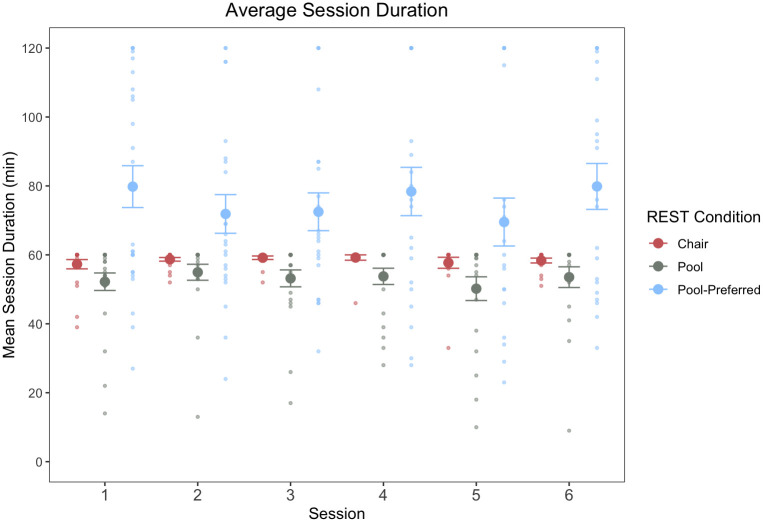
Session durations. *Note*. Data reflects mean and standard error of session durations for each visit by REST condition, as well as individual session durations (individual data points) depicted in lighter shades. Session durations were significantly longer for the pool-REST preferred condition (*M* = 75.4 minutes, *SD* = 29.4) than either the chair-REST (*M* = 58.4 minutes, *SD* = 4.3, *p* = .0003, *d* = .810, 95% CI [-30.42, -4.75]) or pool-REST condition (*M* = 53.0 minutes, *SD* = 12.3; *p* = .003, *d* = .996, 95% CI [-36.98, -10.77]). There were no statistically significant differences in session duration between the chair-REST and pool-REST conditions, which had prescribed session durations of 60 minutes (*p* = .23, 95% CI [-6.44, 19.02]). Duration did not vary as a function of session number.

*Session duration*. The linear mixed effects analysis of within-session tolerability (i.e., session duration; Fig 6), revealed a main effect of condition (F(2) = 10.33, *p* < .001, η_p_^2^ = .24), such that session durations were significantly longer for the pool-REST preferred condition (*M* = 75.4 minutes, *SD* = 29.4) than either the chair-REST (*M* = 58.4 minutes, *SD* = 4.3, *p* = .0003, *d* = .810, 95% CI [-30.42, -4.75]) or pool-REST condition (*M* = 53.0 minutes, *SD* = 12.3; *p* = .003, *d* = .996, 95% CI [-36.98, -10.77]). There were no statistically significant differences in session duration between the chair-REST and pool-REST conditions, which had prescribed session durations of 60 minutes (*p* = .23, 95% CI [-6.44, 19.02]). Across the 373 REST sessions administered across the entire study, 317 sessions (85% of total) were 50 minutes or longer in duration.

*REST preference*. When allowed to select the frequency of sessions, the pool-REST preferred participants chose to float at a frequency of once every 12 days, on average (*SD* = 9.33), and for a mean float duration of 75 minutes (*M* = 75.4 minutes, *SD* = 29.4). Exploratory analyses indicated that session frequency and session duration were not significantly associated with baseline measures of anxiety, depression, or anxiety sensitivity (*p*s >.05).

### Safety

*Adverse events*. No serious adverse events were recorded during the study. Four adverse events were recorded during the study. All were determined to be unrelated to the study intervention. One participant in the chair-REST condition reported two separate instances of migraines with nausea on the days of their first and second REST session. One participant in the pool-REST condition who suffered from seasonal allergies reported an ear infection four days after their third REST session. Finally, one participant in the pool-REST condition reported a suicide attempt the night before their 6-week follow-up visit. The participant was subsequently evaluated at a local psychiatric emergency department, where it was determined that hospital admission was unwarranted.

**Fig 7:**
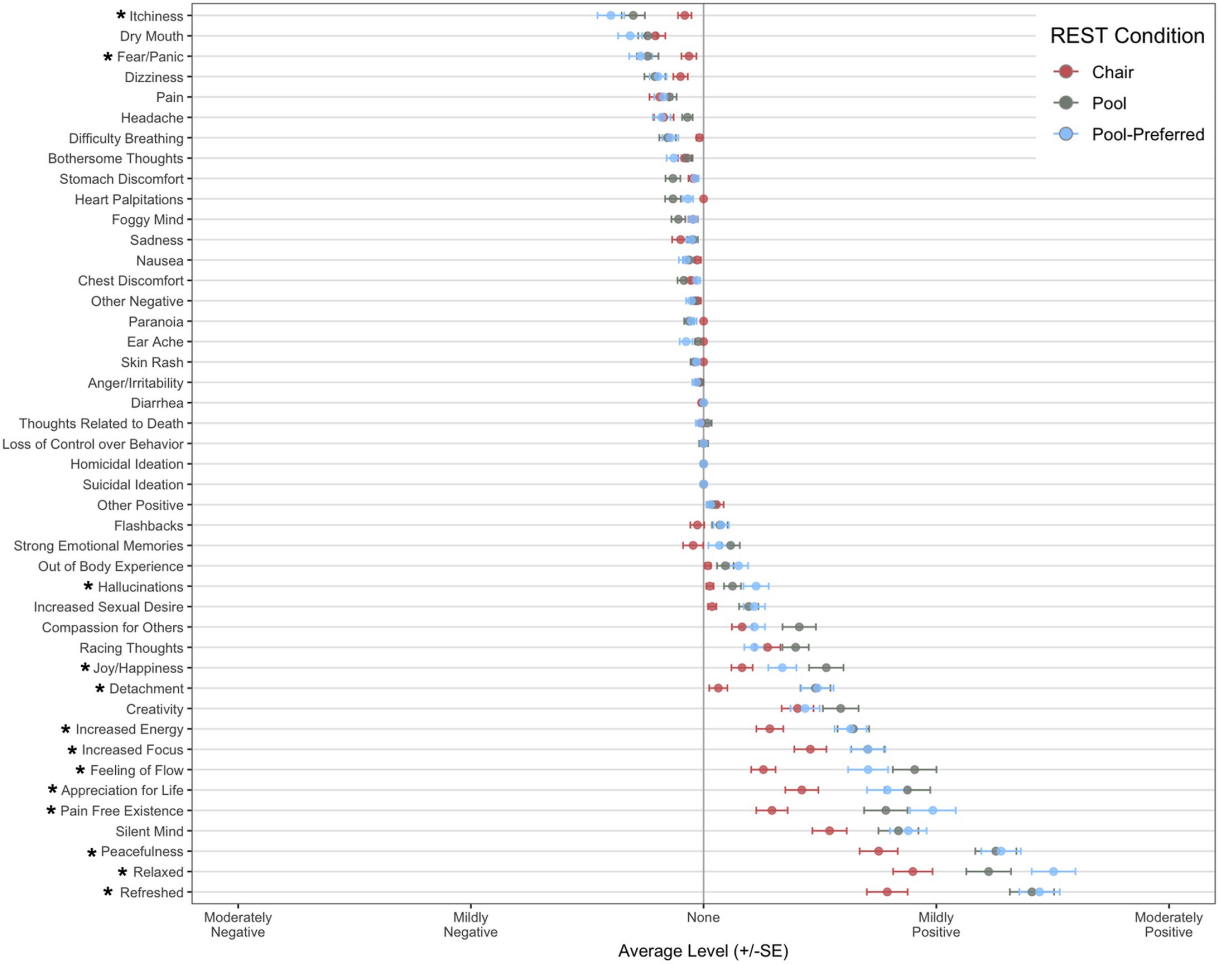
Events assessment. Note. Data reflects mean and standard error of magnitude ratings for each event by REST condition. Event anchors ranged from “Extremely Negative” to “Extremely Positive” (anchors not shown). Asterisk (*) indicates significant group difference in rating magnitude.

*Events assessment*. Regarding the acutely experienced event profile, Fig 7 shows the mean magnitude rating for each event across the three assigned REST conditions. A qualitative inspection of the data revealed that positive experiences were endorsed more commonly than negative ones and were also rated at higher levels of magnitude, with average intensity ratings in the mild-to-moderate range for positive experiences and well-below mild for negative experiences. Across the entire study, a total of 79 experiences were rated in the moderately negative range (S1 Fig), and a total of 11 experiences were rated as extremely negative (S2 Fig). In the linear mixed effects (LME) quantitative analysis of intensity ratings, we observed a significant main effect of event (F(43) = 153.61, *p* < .001, η_p_^2^ = .30) and treatment condition (F(2) = 3.51, *p* = .04, η_p_^2^ = .09), which was accounted for by the significant interaction between these variables (F(86) = 6.43, *p* < .0001, η_p_^2^ = .04). Post-hoc comparisons suggested that feelings of intense joy/happiness, increased energy, increased ability to focus/concentrate, serenity and peacefulness, appreciation for life, refreshment, relaxation, silent mind, pain-free existence, and feelings of flow were experienced more positively in the pool-REST and pool-REST preferred conditions when compared to the chair-REST condition (*p*s < .05). Stronger positive ratings for the pool-REST preferred condition were also observed for relaxation and pain-free existence when compared to the pool-REST condition (*p*s < .05). The opposite trend was found for feelings of flow, increased empathy and compassion for others, and joy/happiness, such that participants in the pool-REST condition tended to rate these items more positively than their pool-REST preferred counterparts (*p*s < .05). The pool-REST group also rated feelings of empathy and compassion for others more positively than their chair-REST counterparts (*p* < .01).

Regarding events that typically carry negative connotations, post-hoc comparisons suggested that itchiness was experienced more negatively in the pool-REST and pool-REST preferred conditions than the chair-REST condition (*p*s < .05). The pool-REST preferred condition also rated feelings of intense fear and panic more negatively than the chair-REST condition (*p* = .045). Interestingly, the pool-REST and pool-REST preferred conditions endorsed experiencing feelings of detachment more positively than the chair-REST condition (*p*s < .001). Stronger positive ratings were also observed in the pool-REST preferred condition than the chair-REST condition for hallucinations (*p* = .03). Of note, the reports of hallucinations and feelings of detachment occurred infrequently and tended to be described in neutral or positive terms. For example, descriptions of hallucinations commonly mentioned visually experienced colors and shapes, such as “*Colorful visions*, *shapes*”, “*Saw green*, *crystal-like geometric shapes off and on (eyes were closed)*”, and “*I experienced those colorful flashes and dots similar to when you close your eyes and press lightly on your eyes*.” Feelings of detachment were typically described using feelings of relaxation, weightlessness, and disconnection from space and time, including: “*I felt weightless*. *I felt like nothing else mattered*. *It was just me*”, “*Felt very relaxed like everything was far away*”, “*A similar dozing detachment as when you’re half asleep for a really nice nap*”, and “*I felt like I was in my own world and the real world was frozen in time*.”

The linear mixed model analysis also revealed a significant interaction between event and visit (F(214) = 1.40, *p* = .0001, η_p_^2^ = .02). Specifically, post-hoc analyses showed a weakening in the magnitude of positive events for all three interventions over the course of the study for the following events: serenity and peacefulness, appreciation for life, refreshment, relaxation, silent mind, pain-free existence, and feelings of flow (*p*s < .05).

## Discussion

This study represents the first randomized controlled trial examining the feasibility, tolerability, and safety of repeated sessions of floatation-REST in individuals with anxiety and depression. We observed evidence of a favorable feasibility, tolerability, and safety profile for the application of pool-REST in this outpatient sample, and acceptable tolerability and safety for chair-REST. Positive experiences were endorsed more commonly than negative ones and were also rated at higher levels of intensity, with no serious adverse events associated with any intervention. Taken together, six sessions of floatation-REST appear feasible, well-tolerated, and safe in anxious and depressed outpatient individuals.

Our primary outcome of adherence suggested that the feasibility of pool-REST, indexed via adherence to the allocated six-sessions, was adequate, ranging from 85–89% for the pool-REST conditions, whereas adherence to the chair-REST condition, at 74%, was not adequate as it fell below the hypothesized 80% adherence rate. It should be noted that due to the limited sample size (and thus limited power) of this preliminary pilot study, no between-group analyses were conducted to statistically assess for between-group differences in adherence. As such, it remains uncertain the extent to which the observed differences in adherence rates were statistically or clinically meaningful. Notwithstanding this limitation, these levels of adherence are broadly comparable to traditional behavioral interventions for anxiety and depression, whose treatment completion rates hover around 80% [30]. Each REST intervention was perceived to be credible, with participants having positive expectations for symptom improvement. The observed levels of credibility and expectancy beliefs are also consistent with those typically seen for other behavioral interventions such as exposure therapy (range of credibility beliefs = 5.95–7.47, range of expectancy beliefs = 50.50–67.90; [40, 44, 49]).

The low dropout rates in this behavioral intervention study provides additional evidence that repeated sessions of floatation-REST are well-tolerated. Dropout rates in the post randomization setting prior to intervention onset were lowest for the pool interventions (4% dropout for pool-REST [n = 1], and 0% dropout for pool-REST preferred [n = 0]), while the chair-REST pretreatment dropout rate was the highest (16%, n = 4) and similar to those observed to cognitive behavioral therapy (dropout rates ranging from 11–21%, [29]). Dropout rates during the administration of the floatation-REST intervention (32% for chair-REST [n = 9], 24% for pool-REST [n = 6], and 16% for pool-REST preferred [n = 4]) were also comparable to rates seen in non-pharmacological and pharmacological interventions such as cognitive behavioral therapy (19–36%, [29]), meditation (33–44%, [50]), acceptance and commitment therapy (15%, [51]), and antidepressant medications (37–49%, [52]). Taken together, the higher adherence rates and lower dropout rates in the pool-REST conditions in comparison to chair-REST, raise the possibility of greater feasibility and tolerability, and perhaps even preference, suggesting pool-REST’s potential utility in a full-scale clinical trial, although further studies in larger samples would be needed to evaluate these notions using appropriately powered statistical analyses.

Using session duration as an additional proxy of intervention tolerability, for chair-REST and pool-REST conditions who had prescribed session durations of 60 minutes, both groups demonstrated mean session durations near 60 minutes. Additionally, we saw very few individuals in the pool-REST and chair-REST conditions terminate early, with the majority of individuals utilizing their full time allotment. For the pool-REST preferred group, who was given greater flexibility in session duration (i.e., allowed to float for up to two hours), we observed longer mean session durations, such that they tended to float about 25% longer than the prescribed session conditions on average. Moreover, session duration was unrelated to symptom severity at baseline, indicating that individuals with higher levels of anxiety, depression, or anxiety sensitivity were no more or less likely to utilize longer durations (i.e., in a self-medicating or symptom-dependent fashion). Collectively, these findings suggest that a 60-minute REST session is a suitable duration for future clinical trials in a clinically anxious and depressed sample. However, a longer session duration (e.g., 75 minutes) may confer some added benefits in terms of participant preference and the duration of the post-REST effect [19].

The safety findings from this floatation-REST trial are consistent with previous single-session studies of pool-REST and chair-REST which found few negative events and no serious adverse events [10, 12, 28]. Our study expands upon this work by showing a favorable safety profile across repeated sessions. We also demonstrated that positive experiences were endorsed more commonly than negative ones, and at higher levels of intensity, in the mild-to-moderate range. Importantly, while all three intervention groups consistently rated positive effects of the intervention, the pool-REST groups tended to rate positive events more strongly, especially regarding feelings of peace and tranquility. These findings provide support for the overall safety of REST delivered in a pool or chair setting. Moreover, the comparable intensity ratings of many negative events but weaker ratings of positive events within the chair-REST condition suggests that the zero-gravity chair is a suitable active comparator.

There were differential event profiles between the pool-REST conditions and the chair-REST condition, such that participants in the pool conditions reported stronger feelings of tranquility (e.g., refreshment, peacefulness, serenity) than those in the chair-REST condition and tended to rate itchiness and fear/panic more negatively than their chair-REST counterparts. The most common negative events associated with pool-REST were itchiness and dry mouth, which is consistent with our previous pilot study [10], and may be related to the high salt concentration in the pools. In addition, several experiences that traditionally carry negative connotations, including audiovisual hallucinations and feelings of detachment, tended to be described in a positive light in the pool conditions. We do not believe the hallucinations reported by participants in this study reflect hallucinations in the true sense of the term and do not consider them indicative of psychosis-spectrum symptoms. Rather, these visual and auditory sensations may be the result of the brain ‘filling in the gaps,’ i.e., generating sensory representations in the face of absent exteroceptive input to the nervous system. Finally, the repeated nature of this intervention allowed for a thorough examination of event profiles over time. An attenuation in the magnitude of several positive events was observed at later sessions for all three interventions, raising the possibility that the increased familiarity may reduce the novelty of the intervention and the magnitude of positive events.

This study has several limitations and future considerations. First, four individuals in the chair-REST condition and one participant in the pool-REST condition withdrew from the study after randomized allocation to their respective intervention and did not complete the CEQ. These dropouts may suggest the presence of unmet expectations, particularly for the individuals allocated to the chair-REST condition given that the largest dropout occurred in this group. Alternatively, pre-intervention anticipatory anxiety could have played a role in withdrawal, although we did not receive such reports from participants. To avoid the limitation of missing data in the future, subsequent studies should aim to collect the CEQ measure immediately following randomization. Another limitation of the current study is the modest sample size. While our sample size was consistent with those used in previous floatation-REST trials, larger groups may be needed for an adequately powered survival analysis for the purposes of tolerability assessment. As such, the current study may be underpowered to detect meaningful group differences in dropout rate. We examined six-session feasibility of REST whereas prior clinical trials have typically used more sessions (e.g., 12). Thus, the measurement of feasibility and tolerability during the employment of a larger number of sessions may be advisable in future trials. It should also be noted that while the measure used to assess for safety (*Events Checklist*) has precedent [10], its psychometric properties have not been formally established. Further, the results from the *Events Checklist* remain somewhat exploratory, as we did not have strong, prespecified hypotheses as to which of the 43 items/events would be differentially impacted by various forms of REST. Additionally, most of our sample was comprised of Caucasian, highly educated females, thereby limiting generalizability claims to the broader population. Finally, due to the fact that participants were financially compensated for their participation, we did not examine structural aspects of feasibility relevant to the real-world delivery of behavioral therapies, including financial and time commitments that might otherwise be important for treatment engagement.

## Conclusion

The findings from this clinical trial suggests that six sessions of floatation-REST are feasible, well-tolerated, and safe in clinically anxious and depressed outpatient individuals. Larger randomized controlled trials evaluating markers of clinical efficacy are warranted.

## Supporting information

S1 Table Event by visit interaction post-hoc comparisons. (PDF)

S2 Table Event by condition interaction post-hoc comparisons. (PDF)

S1 Fig Frequency of events rated moderately negative by REST condition. (PDF)

S2 Fig Frequency of events rated extremely negative by REST condition. (PDF)

S1 Checklist CONSORT 2010 checklist of information to include when reporting a pilot or feasibility trial*. (DOC)

S1 File (DOCX)
