# Supplementary material for: A randomized controlled safety and feasibility trial of floatation-REST in anxious and depressed individuals
Source: PLoS One. 2024 Jun 6;19(6):e0286899. doi: 10.1371/journal.pone.0286899 (PMC11156321; doi:10.1371/journal.pone.0286899)
Supplement: S1 Table — (PDF) [file pone.0286899.s001.pdf]

Supplemental Table 1. Event by Visit Interaction Post-hoc Comparisons.

	Comparison							
	Visit 1 vs. 2		Visit 1 vs. 3		Visit 1 vs. 4		Visit 1 vs. 5	
Event	*p*	*d*	*p*	*d*	*p*	*d*	*p*	*d*
Serenity/Peacefulness	1.00	0.25	1.00	0.133	< 0.01	0.68	0.13	0.46
Life Appreciation	0.82	0.31	0.26	0.41	0.02	0.58	0.40	0.38
Refreshed	1.00	0.00	1.00	0.25	0.05	0.52	1.00	0.13
Relaxed	1.00	0.18	0.29	0.39	0.05	0.52	< 0.01	0.64
Silent Mind	< 0.01	0.63	< 0.01	0.95	< 0.01	1.08	< 0.01	1.06
Pain Free Existence	0.85	0.28	< 0.01	0.83	0.43	0.36	1.00	0.26
Feelings of Flow	< 0.01	0.81	< 0.01	0.85	< 0.01	0.61	< 0.01	0.85
Dizziness	1.00	0.01	0.46	0.36	0.84	0.29	0.04	0.54

Note. p indicates p-value associated with post-hoc comparison test of simple effects. *d* represents associated Cohen's D effect size.
