# Supplementary material for: A randomized controlled safety and feasibility trial of floatation-REST in anxious and depressed individuals
Source: PLoS One. 2024 Jun 6;19(6):e0286899. doi: 10.1371/journal.pone.0286899 (PMC11156321; doi:10.1371/journal.pone.0286899)
Supplement: S2 Table — (PDF) [file pone.0286899.s002.pdf]

Supplemental Table 2. Event by Condition Interaction Post-hoc Comparisons.

	Comparison					
	Chair-REST vs. Pool-REST		Chair-REST vs. Pool-REST Preferred		Pool-REST vs. Pool-REST Preferred	
Event	*p*	*d*	*p*	*d*	*p*	*d*
Joy/Happiness	< 0.01	0.69	0.04	0.34	0.04	0.35
Increased Energy	< 0.01	0.68	< 0.01	0.67	0.98	0.00
Increased Focus	< 0.01	0.46	< 0.01	0.49	0.85	0.03
Serenity/Peacefulness	< 0.01	0.94	< 0.01	1.01	0.63	0.07
Empathy/Compassion	0.01	0.47	0.42	0.13	0.04	0.35
Life Appreciation	< 0.01	0.85	< 0.01	0.70	0.29	0.16
Refreshed	< 0.01	1.17	< 0.01	1.25	0.58	0.08
Relaxed	< 0.01	0.61	< 0.01	1.16	< 0.01	0.55
Silent Mind	< 0.01	0.55	< 0.01	0.64	0.53	0.09
Pain Free Existence	< 0.01	0.94	< 0.01	1.34	< 0.01	0.40
Feelings of Flow	< 0.01	1.24	< 0.01	0.87	0.01	0.37
Fear/Panic	0 .08	0.33	0.05	0.38	0.71	0.05
Itchiness	0.02	0.42	< 0.01	0.60	0.22	0.18
Hallucinations	0.31	0.19	0 .03	0.40	0.31	0.21
Detachment	< 0.01	0.78	< 0.01	0.81	0.82	0.03

Note. p indicates p-value associated with post-hoc comparison test of simple effects. *d* represents associated Cohen's D effect size
