# Supplementary material for: A randomized controlled safety and feasibility trial of floatation-REST in anxious and depressed individuals
Source: PLoS One. 2024 Jun 6;19(6):e0286899. doi: 10.1371/journal.pone.0286899 (PMC11156321; doi:10.1371/journal.pone.0286899)
Supplement: S2 Fig — (PDF) [file pone.0286899.s004.pdf]

Supplemental Figure 2. Frequency of Events Rated Extremely Negative by REST Condition.

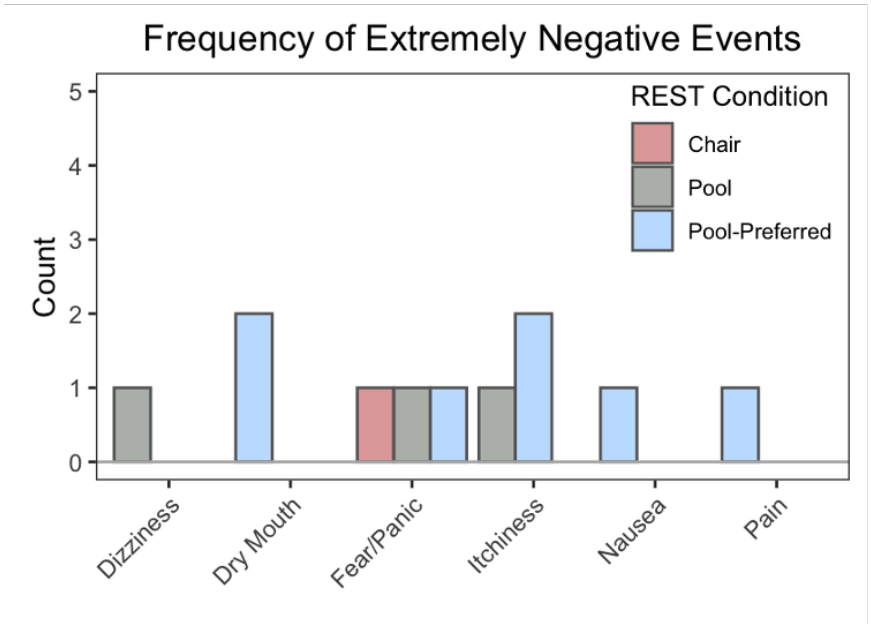

Note. Counts are collapsed across all six REST sessions.
