# Supplementary material for: A randomized controlled safety and feasibility trial of floatation-REST in anxious and depressed individuals
Source: PLoS One. 2024 Jun 6;19(6):e0286899. doi: 10.1371/journal.pone.0286899 (PMC11156321; doi:10.1371/journal.pone.0286899)
Supplement: S1 File — (DOCX) [file pone.0286899.s006.docx]

**Supplemental Materials**

***Events Checklist***

**Instructions**: Did you notice or experience an INCREASE in any of the items below during or shortly after your float today? Please only mark items that showed an increase from your typical day-to-day experience.

**Items**:

1. Heightened creativity
2. Flight of ideas or racing thoughts
   1. Overall, was this a positive or negative experience?
3. Feelings of intense euphoria, joy, or happiness
4. Heightened energy
5. Heightened focus and ability to concentrate
6. A feeling of total serenity and peacefulness
7. Increased sexual desire
8. Heightened empathy and compassion for others
9. A strong feeling of appreciation that you are alive
10. Feeling completely refreshed, like the reset button was hit
11. Total relaxation of the body (without any muscle tension)
12. Complete silence of mind (without any anxious thoughts or worries)
13. Totally pain-free existence
14. A feeling of “flow” with the world around you
15. Dizziness
16. Headache or migraine
17. Feelings of intense fear, anxiety, or panic
18. Difficulty breathing
19. Heart palpitations
20. Chest discomfort
21. Stomach discomfort
22. Nausea
23. Diarrhea
24. Pain
25. Itchiness
26. Skin rash
27. Dry mouth
28. Ear ache
29. Strong emotional memories
    1. Overall, was this a positive or negative experience?
30. Flashbacks (reliving a memory as if it were happening all over again)
    1. Overall, was this a positive or negative experience?
31. Heightened thoughts related to death
    1. Overall, was this a positive or negative experience?
32. A desire or wanting to hurt or kill yourself
33. A desire or wanting to hurt or kill others
34. Visual or auditory hallucinations
    1. Overall, was this a positive or negative experience?
35. Paranoia (intense fear or suspicion of others)
36. Out-of-Body experiences
    1. Overall, was this a positive or negative experience?
37. Feeling detached from the world around you
    1. Overall, was this a positive or negative experience?
38. Loss of control over behavior
    1. Overall, was this a positive or negative experience?
39. Sadness or hopelessness
40. Foggy or cloudy mind
41. Heightened degree of anger or irritability
42. Bothersome or worrisome thoughts
43. Other
    1. Overall, was this a positive or negative experience?

**Response Anchors:** None, Mild, Moderate, Extreme

***Instructions for Floatation-REST Sessions***

The following instruction set was provided to all individuals prior to their floatation-REST session for both chair-REST and pool-REST conditions: *“Thank you so much for your help in participating in this research study. Our goal is to learn more about the effects of reducing environmental stimulation on the nervous system. Throughout the day, our brain is constantly bombarded by sensory information from the external world. In this study, we aim to understand what happens when the brain gets a chance to disconnect from this constant stimulation by floating in an environment with reduced levels of light and sound, and reduced pressure on the spinal cord. While floating, try to remain still. It’s okay if you move, but just try your best not to move too much. Also, try your best not to fall asleep. We realize that you might fall asleep on occasion, but it’s important to keep in mind that our study is focused on what happens to the brain while you are awake. While most prefer to float with the lights off, the choice is yours and you are in complete control the entire time. You can float for up to 60 minutes and you are always free to stop at any time. We will turn on some music after an hour has passed. Take your time getting up, there is no rush. Before we begin, do you have any questions?”* The pool-REST preferred condition instruction set was modified to read, *“You can float for up to 120 minutes and you are always free to stop at any time. We will turn on some music after two hours have passed.”*

***Floatation-REST Environmental Preference***

For both the pool-REST and pool-REST preferred conditions, for each session participants were allowed to select from either an open or enclosed pool, and the choice frequency was calculated for each pool condition. To determine if there were differential pool preferences (open vs. enclosed pool) between the experimental conditions (pool-REST vs. pool-REST preferred), a 2x2 chi-squared test was conducted. The pool-REST condition chose to float in the open pool for 45.7% of their sessions, and the enclosed pool for the remaining 54.3% of their sessions. The pool-REST preferred participants followed a similar pattern, choosing the open pool 54.9% of the time, and choosing the enclosed pool 45.1% of the time. There were no significant differences in pool preference between the pool-REST and pool-REST preferred conditions (*X^2^* =1.85, *p* = 0.17).

***Serious Adverse Event (SAE) Definition***

A SAE is an AE which meets any of the following criteria:

* Results in death

* Is life-threatening

* Requires hospitalization or the prolongation of existing hospitalization

* Results in a persistent or significant disability or incapacity

* Is a congenital anomaly or birth defect

The term "life threatening" refers to an event in which the patient is at risk of death at the time of the event; it does not refer to an event, which hypothetically might have caused death if it were more severe.

Medical and scientific judgment should be exercised in deciding whether an AE is serious. Some important medical events that may not result in death, be life threatening, or require hospitalization may be considered SAEs when they may jeopardize the patient such that medical or surgical intervention is needed to prevent 1 of the outcomes previously listed. Examples of such medical events include intensive emergency treatment for an allergic reaction, blood dyscrasias or convulsions that do not result in inpatient hospitalization, and the development of drug dependency or drug abuse.

Visits to urgent care or emergency room facilities may not warrant reporting as SAEs unless the patient is admitted to the hospital or the event meets other "serious" criteria. Events that are not clearly meeting "serious" criteria can be discussed on a case by case basis with the medical monitor to help the Investigator determine whether the event meets "serious" criteria.

If either the Sponsor or principal Investigator believes that any event is serious, the event must be considered and evaluated by the Sponsor for possible expedited reporting.

Clarification of the difference between "serious" and "severe":

The term "severe" is often used to describe the intensity (severity) of a specific event (as in mild, moderate, or severe myocardial infarction); the event itself, however, may be of relatively minor medical significance (such as a severe headache). This is not the same as "serious," which is based on patient/event outcome or action criteria usually associated with events that pose a threat to a patient's life or functioning. Seriousness (not severity) serves as a guide for defining regulatory reporting obligations.

*Supplemental Table 1.* Event by Visit Interaction Post-hoc Comparisons.

|  | Comparison | | | | | | | | | |
| --- | --- | --- | --- | --- | --- | --- | --- | --- | --- | --- |
|  | Visit 1 vs. 2 | | Visit 1 vs. 3 | | Visit 1 vs. 4 | | Visit 1 vs. 5 | | Visit 1 vs. 6 | |
| *Event* | *p* | *d* | *p* | *d* | *p* | *d* | *p* | *d* | *p* | *d* |
| Serenity/Peacefulness | 1.00 | 0.25 | 1.00 | 0.133 | < 0.01 | 0.68 | 0.13 | 0.46 | 0.05 | 0.54 |
| Life Appreciation | 0.82 | 0.31 | 0.26 | 0.41 | 0.02 | 0.58 | 0.40 | 0.38 | < 0.01 | 0.66 |
| Refreshed | 1.00 | 0.00 | 1.00 | 0.25 | 0.05 | 0.52 | 1.00 | 0.13 | 0.02 | 0.59 |
| Relaxed | 1.00 | 0.18 | 0.29 | 0.39 | 0.05 | 0.52 | < 0.01 | 0.64 | < 0.01 | 0.66 |
| Silent Mind | < 0.01 | 0.63 | < 0.01 | 0.95 | < 0.01 | 1.08 | < 0.01 | 1.06 | < 0.01 | 0.88 |
| Pain Free Existence | 0.85 | 0.28 | < 0.01 | 0.83 | 0.43 | 0.36 | 1.00 | 0.26 | 0.48 | 0.35 |
| Feelings of Flow | < 0.01 | 0.81 | < 0.01 | 0.85 | < 0.01 | 0.61 | < 0.01 | 0.85 | < 0.01 | 0.83 |
| Dizziness | 1.00 | 0.01 | 0.46 | 0.36 | 0.84 | 0.29 | 0.04 | 0.54 | 0.25 | 0.42 |

*Note. p* indicates p-value associated with post-hoc comparison test of simple effects. *d* represents associated Cohen’s D effect size.

*Supplemental Table 2.* Event by Condition Interaction Post-hoc Comparisons.

|  | Comparison | | | | | |
| --- | --- | --- | --- | --- | --- | --- |
|  | Chair-REST vs.  Pool-REST | | Chair-REST vs.  Pool-REST Preferred | | Pool-REST vs.  Pool-REST Preferred | |
| *Event* | *p* | *d* | *p* | *d* | *p* | *d* |
| Joy/Happiness | < 0.01 | 0.69 | 0.04 | 0.34 | 0.04 | 0.35 |
| Increased Energy | < 0.01 | 0.68 | < 0.01 | 0.67 | 0.98 | 0.00 |
| Increased Focus | < 0.01 | 0.46 | < 0.01 | 0.49 | 0.85 | 0.03 |
| Serenity/Peacefulness | < 0.01 | 0.94 | < 0.01 | 1.01 | 0.63 | 0.07 |
| Empathy/Compassion | 0.01 | 0.47 | 0.42 | 0.13 | 0.04 | 0.35 |
| Life Appreciation | < 0.01 | 0.85 | < 0.01 | 0.70 | 0.29 | 0.16 |
| Refreshed | < 0.01 | 1.17 | < 0.01 | 1.25 | 0.58 | 0.08 |
| Relaxed | < 0.01 | 0.61 | < 0.01 | 1.16 | < 0.01 | 0.55 |
| Silent Mind | < 0.01 | 0.55 | < 0.01 | 0.64 | 0.53 | 0.09 |
| Pain Free Existence | < 0.01 | 0.94 | < 0.01 | 1.34 | < 0.01 | 0.40 |
| Feelings of Flow | < 0.01 | 1.24 | < 0.01 | 0.87 | 0.01 | 0.37 |
| Fear/Panic | 0 .08 | 0.33 | 0.05 | 0.38 | 0.71 | 0.05 |
| Itchiness | 0.02 | 0.42 | < 0.01 | 0.60 | 0.22 | 0.18 |
| Hallucinations | 0.31 | 0.19 | 0 .03 | 0.40 | 0.31 | 0.21 |
| Detachment | < 0.01 | 0.78 | < 0.01 | 0.81 | 0.82 | 0.03 |

*Note. p* indicates p-value associated with post-hoc comparison test of simple effects. *d* represents associated Cohen’s D effect size

Supplemental Figure 1. Frequency of Events Rated Moderately Negative by REST Condition

**
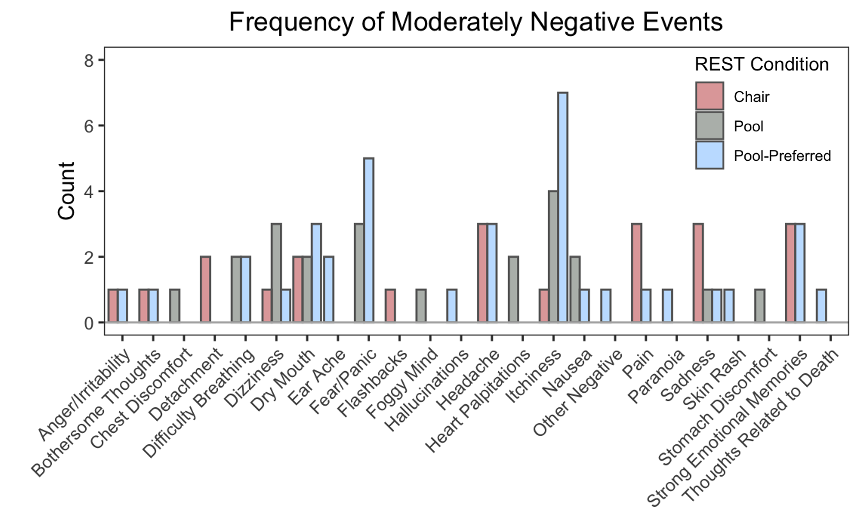
**

*Note.* Counts are collapsed across all six REST sessions.

Supplemental Figure 2. Frequency of Events Rated Extremely Negative by REST Condition

**
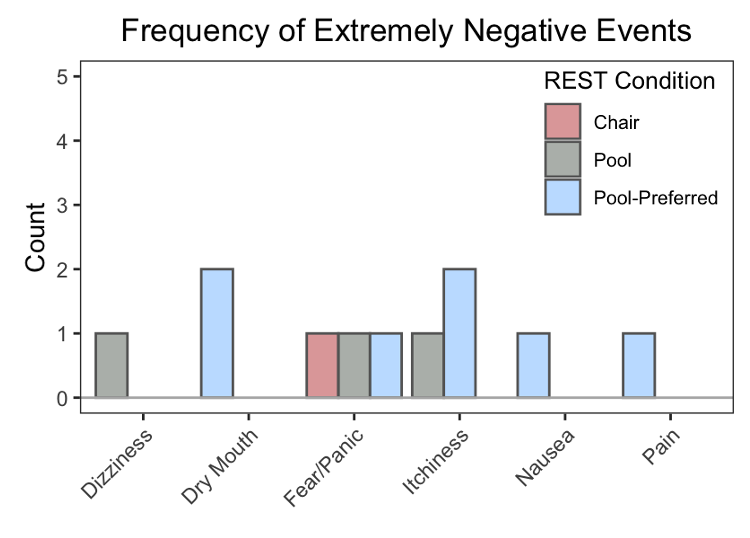
**

*Note.* Counts are collapsed across all six REST sessions.
